# Alcohol consumption and allergic diseases: Mendelian randomization evidence from China

**DOI:** 10.1080/16549716.2024.2442788

**Published:** 2025-01-22

**Authors:** Chen Zhu, Timothy Beatty, Yingxiang Li, Gang Chen, Qiran Zhao, Qihui Chen

**Affiliations:** a College of Economics and Management, China Agricultural University, Beijing, China; b Beijing Food Safety Policy & Strategy Base, China Agricultural University, Beijing, China; c Department of Agricultural and Resource Economics, University of California, Davis, USA; d WeGene, Shenzhen Zaozhidao Technology Co. Ltd., Shenzhen, China; e Graduate Affairs, Faculty of Medicine, Chulalongkorn University, Bangkok, Thailand

**Keywords:** Alcohol drinking, allergic diseases, causal effects, genetic instrumental variables, gender differences

## Abstract

**Background:**

The prevalence of allergic diseases in China has risen significantly over the past decades, affecting the quality of life for approximately 40% of the population.

**Objectives:**

This study aimed to integrate survey and genomic data to explore the potential causal relationship between alcohol consumption and allergic diseases.

**Method:**

In collaboration with a leading genetic testing company in China, we collected data on 3,041 participants via an online survey between December 2018 and October 2019. A Mendelian Randomization (MR) design was employed in data analysis, leveraging the random allocation of genes at meiosis in humans to create instrumental variables for alcohol intake. This method was used to estimate the causal effect of alcohol consumption on the incidence of allergic diseases.

**Results:**

While ordinary least-squares estimates showed a negative association between alcohol drinking and the risk of self-reported allergic diseases, MR estimates suggest that higher alcohol consumption increased the risks of allergy in certain subgroups. Specifically, predicted drinking [b = 0.445, *p* = 0.032] and the number of drinking times during the past 30 days [b = 0.031, *p* < 0.01] were associated with higher risks of allergic diseases among females. We found little evidence of a causal impact of alcohol intake on allergic diseases in men.

**Conclusion:**

Higher alcohol intake is causally associated with a higher risk of allergic diseases in Chinese women but not men.

## Paper Context

- **Main findings:** Mendelian Randomization estimates suggest that higher alcohol intake increased the risks of allergy in women but not in men.
- **Added knowledge:** Mendelian Randomization analyses identified the causal link between alcohol consumption and allergic diseases.
- **Global health impact for policy and action:** Provided evidence to inform global alcohol consumption and health policy.

## Background

Allergic diseases refer to any exaggerated immune response to a foreign antigen that may severely impact a patient’s quality of life [1,2]. An increasing occurrence of allergic diseases has been reported in China during the past decades, affecting 40% of the population [3,4]. For example, between 2005 and 2011, the prevalence of allergic rhinitis, atopic dermatitis, and asthma among residents in eighteen major cities in China was estimated to be 17.6%, 14.0%, and 5.8%, respectively [5]. With rapidly rising prevalence rates, allergic diseases have become a public health challenge that might impose a substantial socioeconomic burden on the Chinese population [6–9].

As a potential risk factor, alcohol consumption can trigger allergic reactions in many ways. First, some individuals are allergic to alcohol, with allergic symptoms caused by alcohol intolerance [10,11]. Second, alcohol consumption may impact the immune system, bringing new allergic symptoms or worsening existing ones [12–14]. In particular, alcohol has been documented as a strong immune-modulating factor associated with elevated serum levels of total immunoglobulin E [15–17]. Finally, allergic reactions can be caused by an intolerance to non-alcoholic ingredients in alcoholic drinks, such as the histamines in red wine and the gluten in beer and some hard liquors [18]. A strong positive link between alcohol consumption and risks of specific allergic diseases, such as perennial and seasonal allergic rhinitis, has been reported in many previous studies [19–22]. However, the extent to which these documented relationships are causal remains debatable, as conventional observational studies often fail to derive credible causal inferences due to commonly encountered problems such as reverse causality and unobserved confounding [23].

Because one’s alcohol consumption behavior is usually not randomly determined, addressing the aforementioned threats to causal identification is crucial for assessing the consequences of alcohol consumption. Several recent studies have exploited Mendelian Randomization (MR), a research design that exploits genetic variants to create instrumental variables (IVs) for alcohol intake to estimate its effects [24]. The ‘quasi-experimental’ nature of the MR approach comes from the random allocation of genes at meiosis in humans, which resembles the random assignment into treatment and control groups in randomized controlled trials (RCTs) that may be infeasible or unethical in the setting of alcohol consumption [25,26]. Using *ADH1B* rs1229984 as a genetic IV, Lawlor et al. (2013) [25] and Holmes et al. (2014) [27] identified the effect of alcohol intake on cardiovascular diseases. Focusing on allergic diseases, Skaaby et al. (2019) also used *ADH1B* rs1229948 as the genetic IV for alcohol consumption to identify its causal effect on allergic diseases in a large European sample [28].

However, existing MR applications are not free of concern. First, the proportion of A-allele carriers is relatively low (around 3% in Caucasians), and its connection with alcohol intake is sometimes vague. As such, existing MR studies may suffer from weak IV problems and often require large samples to detect meaningful associations [29–31]. Second, most existing MR studies incorporated genetic IVs in the analysis without validating the key identification assumptions needed, raising concerns about such problems as pleiotropy and dynastic effects that may bias MR estimates [24,32,33].

The present study employs an MR approach to investigate the causal impact of alcohol consumption on the incidence of allergic diseases and explore potential sex-specific patterns in the context of China, a country where the alcohol consumption-allergic diseases relationship has rarely been studied. In applying the MR design, we address the two aforementioned concerns that plagued existing MR studies. First, unlike most existing MR studies, which usually employed one single genetic IV, we employ multiple genetic IVs, *ADH1B* rs1229984 and *ALDH2* rs671 (—the latter only exists in East Asian populations and has strong predictive power for the incidence of alcohol intolerance), to circumvent the weak instrument problem. Second, unlike most existing MR studies, this study is among the first to carefully validate the identification assumptions underlying the MR design. In particular, we control for inferred parental *ALDH2* rs671 genotypes and individuals’ ancestral composition in our MR models to rule out dynastic effects, in addition to control variables commonly used in MR studies, such as individual socioeconomic characteristics, parental drinking behaviors, and regional fixed effects.

Our MR design was applied to data on 3,041 individuals (1,826 males and 1,215 females) collected from the customer database of a leading private genetic testing company in China, WeGene. Given the rising popularity of alcoholic beverages in China [34] and the rising prevalence of allergic diseases across the country [3–9], if the documented link between alcohol consumption and increased risk of allergy is indeed causal, policy implications derived from this link could be substantial from the perspectives of public health and food policy.

## Materials and method

### Data source, sample collection, and variable construction

#### Sample collection

**Figure 1.**
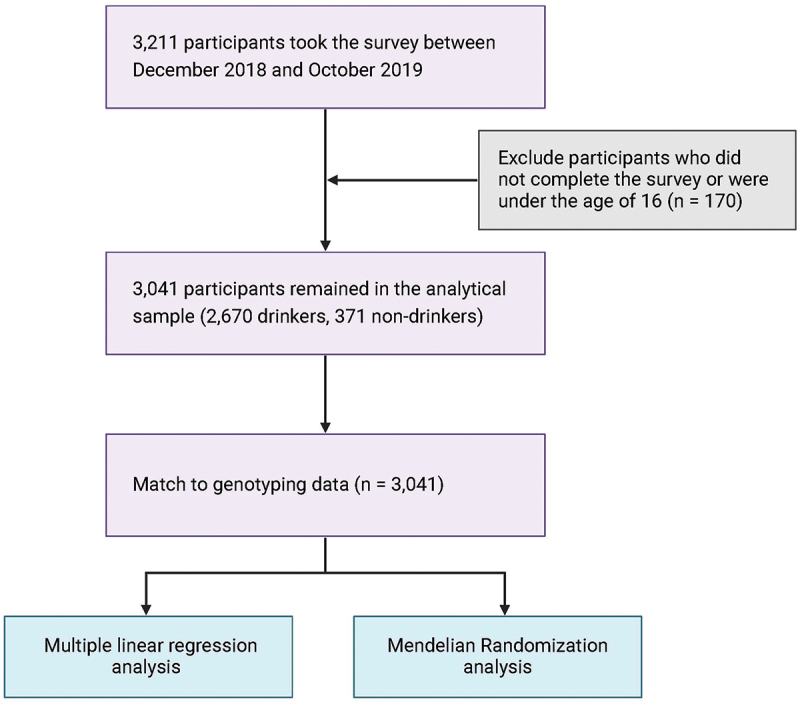
Flowchart of the data collection process and study design.

Our data were collected via a survey designed and conducted by a collaborative effort between the authors and researchers from WeGene, a leading private genetic testing company in China that provides direct-to-consumer (DTC) genetic testing and personalized healthcare services. The Institutional Review Board of China Agricultural University approved the research protocol. A total of 3,211 participants, all from WeGene’s costomer database, took our online survey between December 2018 and October 2019 after providing informed consent. The survey collected information on the participants’ demographic characteristics, socioeconomic status, and alcohol consumption behaviors. A unique feature of the survey is that it also collected data on the participants’ parental alcohol-drinking behaviors (i.e. whether their father/mother drinks). Excluding individuals who did not complete the survey or were under the age of 16 yielded an analytical sample with 3,041 observations (Figure 1). The average respondent in our sample was 28.9 years old, completed 16.3 years of education, and earned about 119,000 CNY (1 US Dollar ≈ 6.5 CNY) annually (Table 1, column 1).

**Table 1.** Summary statistics of the analytic sample.

		(1)	(2)	(3)
Variable		All (*N* = 3,041)	Males (*N* = 1,826)	Females (*N* = 1,215)
*Socio-demographic characteristics:*				
Age (years)		28.9 (8.3)	29.4 (8.8)	28.1 (7.5)
Years of schooling		16.3 (2.0)	16.2 (2.1)	16.5 (2.0)
Annual earnings (in 10,000 CNY)		11.9 (17.2)	13.2 (18.6)	10.0 (14.8)
*Drinking behaviors:*				
Drinking or not				
	Drinkers ( = 1):	87.8%	88.7%	86.5%
	Non-drinkers ( = 0):	12.2%	11.3%	13.5%
Drinking times during the past 30 days		4.0 (6.1)	4.4 (6.4)	3.4 (5.5)
Maximum ethanol consumed on one occasion (in grams)		49.0 (71.1)	60.7 (79.9)	30.9 (49.6)
Alcohol flush after drinking		22.4%	23.0%	20.9%
*Genetic instruments:*				
ALDH2 rs671				
	AA:	2.8%	3.0%	2.5%
	AG:	27.0%	27.1%	26.9%
	GG:	70.2%	69.9%	70.6%
ADH1B rs1229984				
	AA:	46.5%	46.3%	46.8%
	AG:	42.7%	42.6%	42.7%
	GG:	10.8%	11.1%	10.5%
*Health status:*				
Self-assessed health status (from 0= very poor to 5= very good)		3.6 (0.9)	3.6 (0.9)	3.7 (0.9)
Allergic disease		15.0%	13.3%	17.6%
*Drinking behavior of parents:*				
Mother – drinking or not				
	Mother of drinkers:	57.0%	52.2%	64.3%
	Mother of non-drinkers:	43.0%	47.8%	35.7%
Father – drinking or not				
	Father of drinkers:	88.2%	86.4%	90.9%
	Father of non-drinkers:	11.8%	13.6%	9.1%
Mother – alcohol flush after drinking		18.9%	17.5%	20.8%
Father – alcohol flush after drinking		25.4%	23.0%	29.1%

#### Genotyping

DNA extraction and genotyping were performed using the participants’ saliva samples. All participants were genotyped with the Illumina WeGene V2 Array, which includes roughly 700,000 SNPs. Imputation and quality control were carried out using PLINK (1.90 Beta), SHAPEIT (v2.17), and IMPUTE2 (v2.3.1). To ensure data quality, samples and SNPs with genotype call rates below 98.5% were excluded. Pairwise relatedness checks were also conducted: one sample from any pair with an identity-by-descent score greater than 0.125 (indicating a third-degree relative) was removed.

#### Measuring alcohol consumption

To construct the exposure variables of interest, our survey collected information on the respondents’ alcohol consumption behavior based on three complementary measures. The first is a binary indicator of whether a respondent drinks, with 0 denoting non-drinkers (12.2%) and 1 denoting drinkers (87.8%). The second measure is the total number of occasions on which a respondent consumed any alcohol during the past 30 days (mean = 4.0, s.d. = 6.1). The third measure concerns one’s alcohol tolerance. To construct this measure, we calculated the maximum ethanol (i.e. pure alcohol in grams) a respondent consumed on a single occasion during the past 30 days (mean = 49.0, s.d. = 71.1). While self-reported data are often subject to concerns about misreporting, it has been demonstrated that self-reported alcohol consumption during a relatively short recent period suffers less from misreporting when multiple closed-ended questions are used, and the answers to these questions can serve as reliable measures of alcohol consumption [35].

#### Measures of allergic diseases

To collect information on the response variable, we asked the respondents the following question during the survey: ‘Do you have any allergic disease (e.g. asthma, rhinitis, eczema)?’ and then constructed a binary measure of allergic diseases (= 1 if ‘yes’ and 0 if ‘no’) based on their answers. Admittedly, self-reported measures are likely to contain measurement errors and are less accurate than doctor-diagnosed measures, imposing a major limitation on this study. However, self-reported measures can help capture the incidence of allergic diseases for individuals who did not visit the hospital for allergic symptoms (for example, if their symptoms were relatively mild). In fact, many recent allergy studies were conducted based on self-reported measures [5,36,37]. Technically speaking, random measurement errors in the outcome variables (incidence of allergic diseases in our context) will not bias the estimates in linear regression models (e.g. OLS or IV models); they will only increase the variance of the estimates [38].

#### Choices of covariates

To control for the influence of confounding factors, we selected three sets of covariates based on standard theory and previous findings. The first set involves participants’ socio-economic status, including age, gender, years of schooling, and annual income. The second set concerns parental characteristics. More specifically, our models control for participants’ parental drinking behaviors (i.e. whether their father or mother drinks) to capture the potential intergenerational transmission of alcohol drinking habits and the influence of the home environment. Parental *ALDH2* rs671 genotypes, together with 42 estimated compositions of individual ancestry based on each respondent’s genetic data [31,39], are included to strengthen the ‘random gene assignment at meiosis’ condition in MR designs. Finally, province fixed effects are included to capture the influence of unobserved effects of regional alcohol drinking culture.

#### Genetic instrument variables

Two genetic variants are commonly used in MR studies of alcohol intake: the alcohol dehydrogenase 1B gene (*ADH1B* rs1229984) and the aldehyde dehydrogenase 2 gene (*ALDH2* rs671), both of which encode enzymes involved in the metabolic pathway for ethanol and can change the metabolic balance of acetaldehyde in the human body [40]. In the body, ethanol is first converted to acetaldehyde by alcohol dehydrogenase (ADH) and then to acetate by aldehyde dehydrogenase (ALDH).

The enzyme activities of ADH and ALDH are largely determined by the number of effect alleles (A-alleles) in both *ADH1B* rs1229984 and *ALDH2* rs671. In East Asian populations, *ALDH2* rs671 alleles exist with three genotypes, GG (number of A alleles = 0), AG (number of A alleles = 1), and AA (number of A alleles = 2), where the presence of A alleles can significantly decrease the detoxification of acetaldehyde generated during alcohol metabolism in humans as noted above [40,41]. As shown in Table 1, column 1, 29.8% of respondents in our sample are A-allele carriers of *ALDH2* rs671 (i.e. genotypes of AA and AG). Specifically, the proportions of genotypes AA and AG are 2.8% and 27.0%, respectively. In European populations, *ADH1B* rs1229984 has been used as the principal genetic instrument in MR studies of alcohol intake [27]. However, because the proportion of A-allele carriers is very low (around 3% in Europeans), these MR studies require much larger samples. In contrast, a majority of participants are A-allele carriers of the *ADH1B* rs1229984 in our sample (AA: 46.5%; AG: 42.7%), which helps strengthen the power of our MR design.

### Statistical methods

#### Linear regression models

Linear regression models (equation 1) based on ordinary least squares (OLS) techniques were first used to examine the relationship between different measures of alcohol consumption (Alcohol) and the incidence of allergic diseases (Allergy), incorporating sample respondents’ demographic and socio-economic characteristics, parental drinking behavior, ancestral composition, and province fixed effects as covariates (X):

(1) $$\mathrm{Allergy}=\beta_{0}+\beta_{1}\mathrm{Alcohol}+X\beta_{2}+\varepsilon ,$$

where the parameter $\beta_{1}$ captures the effect of alcohol intake on the incidence of allergic diseases, and ε is a disturbance term. For OLS models to yield estimates of $\beta_{1}$ that have causal interpretations, the critical identification assumption needed is that conditional on the set of covariates X, there is no correlation between the disturbance ε and the explanatory variable of primary interest, Alcohol [42].

#### Mendelian randomization analysis

The MR approach estimates the causal impacts of alcohol intake on allergic diseases based on two-stage least squares (2SLS), adjusting for sample respondents’ demographic/socio-economic factors, parental drinking-related traits, ancestral composition, and province fixed effects. More specifically, the following models are estimated in a 2SLS framework. The first-stage model (equation 2) is a linear projection of alcohol intake on the set of genetic IVs, *G* = (*ADH1B* rs1229984, *ALDH2* rs671), and the set of covariates (*Z*) just mentioned:

(2) $$\hat{\mathrm{Alcohol}}={\hat{\alpha }}_{0}+G{\hat{\alpha }}_{1}+Z{\hat{\alpha }}_{2},$$

where $\hat{\mathrm{Alcohol}}$ is the OLS fitted value of the model, and the ${\hat{\alpha }}^{\prime }s$ are the OLS fitted parameters. The second-stage model (equation 3) is similar to equation (1) but with the exposure variable, Alcohol, replaced with its fitted value, $\hat{\mathrm{Alcohol}}$, from equation (2):

(3) $$\mathrm{Allergy}=\eta_{0}+\eta_{1}\hat{\mathrm{Alcohol}}+Z\eta_{2}+\varepsilon .$$

By construction, $\hat{\mathrm{Alcohol}}$ will be uncorrelated with the disturbance ε and its coefficient $\eta_{1}$ will have a causal interpretation if the following three conditions for the genetic IVs (*G*) are met [26,43,44]: (i) *Relevance*: The IVs must be highly correlated with the endogenous exposure variable, Alcohol, after adjusting for the effects of the covariates *Z*; (ii) *Independence*: the genetic IVs should be uncorrelated with unmeasured confounders, conditional on *Z*; (iii) *Exclusion*: controlling for *Z*, the genetic IVs do not have direct effects on health outcomes (allergic diseases) through horizontal pleiotropy. These conditions will be verified below before we present the results of the MR analyses.

Results of estimating both OLS (1) and MR-IV models (2 and 3) are reported below as beta coefficients with standard errors. All p-values were two-sided.

## Results

### Descriptive patterns

#### Sample characteristics

Table 1, columns 1–3, reports the basic characteristics of the full sample, male and female samples, respectively. Column 1 suggests that an average respondent in our analytical sample completed 16.3 years of education and earned 119,000 CNY (1 US Dollar ≈ 6.5 CNY) annually. While both figures are higher than their nationwide counterparts,^1^^1^The average annual wage of an urban employee is 74,318 CNY in 2017 (Source: http://www.chinadaily.com.cn/a/201805/21/WS5b02d4d6a3103f6866ee9b15.html. Accessed: July 15, 2022), and the average educational attainment of an employee is 10.2 years in 2015 (Source: http://www.gov.cn/xinwen/2017-07/25/content_5213292.htm. Accessed: July 15, 2022). they represent a pattern consistent with previous findings: Direct-to-consumer genetic testing customers are generally well-educated middle-class professionals [45]. Columns 2 and 3 further suggest that, on average, female respondents are slightly younger and more educated but earn less than male respondents.

Turning to the exposure variables, the average monthly frequency of drinking and the maximum alcohol intake on one occasion are both higher in men than women. However, the percentages of general drinking behaviors among men (88.7%) and women (86.5%) in our sample are surprisingly comparable. These high percentages are at odds with the findings of Cho et al. (2015) [23], who reported a much lower proportion of female drinkers (25.7%) than male drinkers (72.4%) in a sample of 7,152 participants from South Korea. The higher percentages of current drinkers in our sample could be due to the prevalence of low-alcoholic beverages and a fad of casual drinking among the younger generations in China.^2^^2^For example, it has been reported that young drinkers in China are shifting from traditional Chinese liquors to low-alcoholic drinks, and the percentage of female drinkers born between 1991 and 2000 exceeds that of male drinkers with the same ages. (Source: https://radiichina-com.cdn.ampproject.org/c/s/radiichina.com/chinese-youth-drinking/amp/; https://www.sohu.com/a/427013796_99900352. Accessed: July 15, 2022).

Regarding the response variable, female participants have a higher prevalence (17.6%) of allergic diseases than males (13.3%), although they have similar values of self-assessed health status. It is worth noting that there is no notable difference in the distributions of *ALDH2* rs671 and *ADH1B* rs1229984 genotypes between the two gender groups.

#### Patterns of alcohol intake by ALDH2 rs671 and ADH1B rs1229984 genotypes

**Figure 2.**
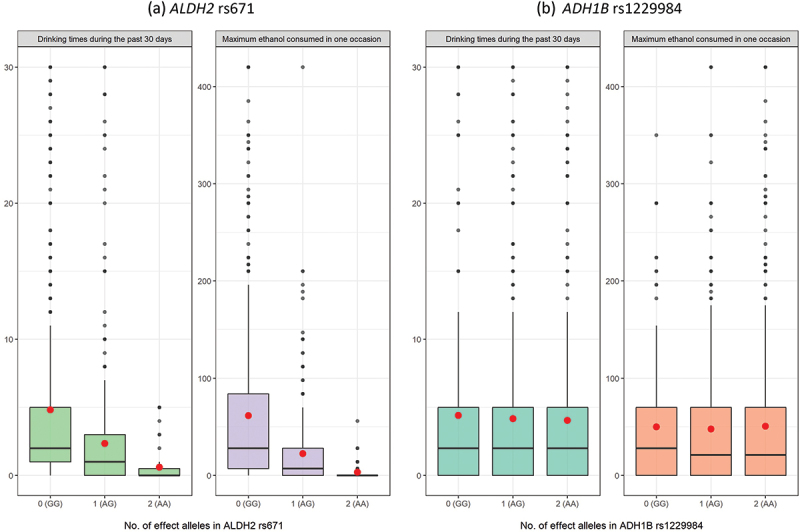
Distributions of alcohol intake by *ALDH2* rs671 and *ADH1B* rs1229984 genotypes.

Note: The numbers of effect A alleles are 0, 1, and 2 in genotypes of GG, AG, and AA, respectively. The solid black lines in boxes represent medians of subsamples. The red dots denote subsample means.

To further examine the correlation between different genotypes and alcohol intake, Figure 2 plots the distributions of drinking frequency and the maximum ethanol consumed on one occasion by *ALDH2* rs671 genotypes (panel A) and *ADH1B* rs1229984 genotypes (panel B), respectively, where solid black lines (in boxes) and red dots denote subsample medians and means. For genetic variations of *ALDH2* rs671 genotypes (panel A), as expected, both subsample medians and means generally drop as the number of A alleles increases from 0 to 2. For genotypes of *ADH1B* rs1229984 (panel B), the association is less clear-cut, and we did not find a monotonically decreasing relationship between alcohol intake and the number of A alleles as in the case of *ALDH2* rs671. This finding is consistent with prior work showing that the association between alcohol consumption and *ADH1B* rs1229984 is weaker than that of *ALDH2* rs671, especially among Chinese populations [29,30,46,47].

### Linear regression results

Table 2 reports parameter estimates of alcohol consumption on allergic diseases from OLS regressions of the full sample (columns 1–3), male participants (columns 4–6), and female participants (columns 7–9), respectively. All estimates are adjusted for the influence of individual demographic characteristics and socio-economic status (i.e. age, gender, years of schooling, and annual income), parental drinking behaviors (i.e. whether one’s father/mother drinks), and province fixed effects.

**Table 2.** Estimated effects of alcohol intake on allergic diseases by linear regressions.

	(1)	(2)	(3)	(4)	(5)	(6)	(7)	(8)	(9)
Variables	All (*N* = 3,041)			Males (*N* = 1,826)			Females (*N* = 1,215)		
Drinking or not	−0.049**			−0.059*			−0.041		
	(0.025)			(0.031)			(0.040)		
Drinking times during the past 30 days		0.001			0.002			−0.000	
		(0.001)			(0.002)			(0.003)	
Maximum number of standard drinks on one occasion			0.002			0.002			0.001
			(0.001)			(0.001)			(0.003)
Male	−0.041**	−0.043***	−0.049***						
	(0.016)	(0.016)	(0.017)						
Age	0.000	−0.000	−0.000	−0.000	−0.001	−0.000	0.000	0.001	0.000
	(0.001)	(0.001)	(0.001)	(0.001)	(0.001)	(0.001)	(0.002)	(0.002)	(0.002)
Annual earnings	0.000	0.000	0.000	0.000	0.000	0.000	−0.000	−0.000	−0.000
	(0.000)	(0.000)	(0.001)	(0.001)	(0.001)	(0.001)	(0.001)	(0.001)	(0.001)
Years of schooling	0.002	0.003	0.002	0.004	0.006	0.004	0.001	0.000	0.001
	(0.004)	(0.004)	(0.004)	(0.005)	(0.005)	(0.005)	(0.007)	(0.007)	(0.007)
Mother drinks	0.021	0.014	0.012	0.043**	0.035*	0.036*	−0.011	−0.016	−0.023
	(0.016)	(0.016)	(0.017)	(0.020)	(0.020)	(0.020)	(0.029)	(0.029)	(0.030)
Father drinks	−0.007	−0.016	−0.014	−0.033	−0.046	−0.043	0.044	0.039	0.040
	(0.025)	(0.025)	(0.025)	(0.029)	(0.029)	(0.029)	(0.048)	(0.048)	(0.050)
Constant	0.182**	0.140*	0.145*	0.143	0.082	0.107	0.155	0.129	0.089
	(0.080)	(0.079)	(0.081)	(0.094)	(0.092)	(0.095)	(0.142)	(0.140)	(0.146)
Province fixed effects	Y	Y	Y	Y	Y	Y	Y	Y	Y
Observations	3,041	3,041	3,041	1,826	1,826	1,826	1,215	1,215	1,215
R-squared	0.023	0.021	0.018	0.026	0.026	0.019	0.054	0.053	0.049

Robust standard errors in parentheses.

***significant at 1% level; **significant at 5% level; *significant at 10% level.

The results show that alcohol drinking is associated with a lower risk of allergic diseases in the full sample [Table 2, column 1: *b* = -0.042, *p* = 0.048] and the male sample [Table 2, column 4: *b* = −0.059, *p* = 0.061]; the associations are statistically significant at least at the 10% level. In contrast, alcohol intake is insignificantly associated with the risk of allergic diseases in women. The sex-specific association between alcohol intake and health outcomes (e.g. blood pressure, HDL cholesterol) has been documented in previous studies [23]. However, it should be noted that the estimated impact of alcohol intake here can still be confounded by various factors (e.g. diet patterns, physical activity, BMI, and insulin resistance levels), even after adjusting for demographic characteristics, socio-economic status, and parental drinking habits. As such, the associations estimated by OLS may not be causal and must be interpreted with caution.

### Mendelian randomization estimates

#### Validity of genetic instrumental variables

Before reporting the results of our Mendelian Randomization analysis, it is helpful to examine the validity of the genetic IVs used first. The validity of these genetic IVs relies on three conditions noted in section 2.2: relevance, independence, and exclusion [26,43,44].

##### Relevance: The IVs must be highly correlated with the endogenous exposure variable (after adjusting for the effects of other covariates)

**Figure 3.**
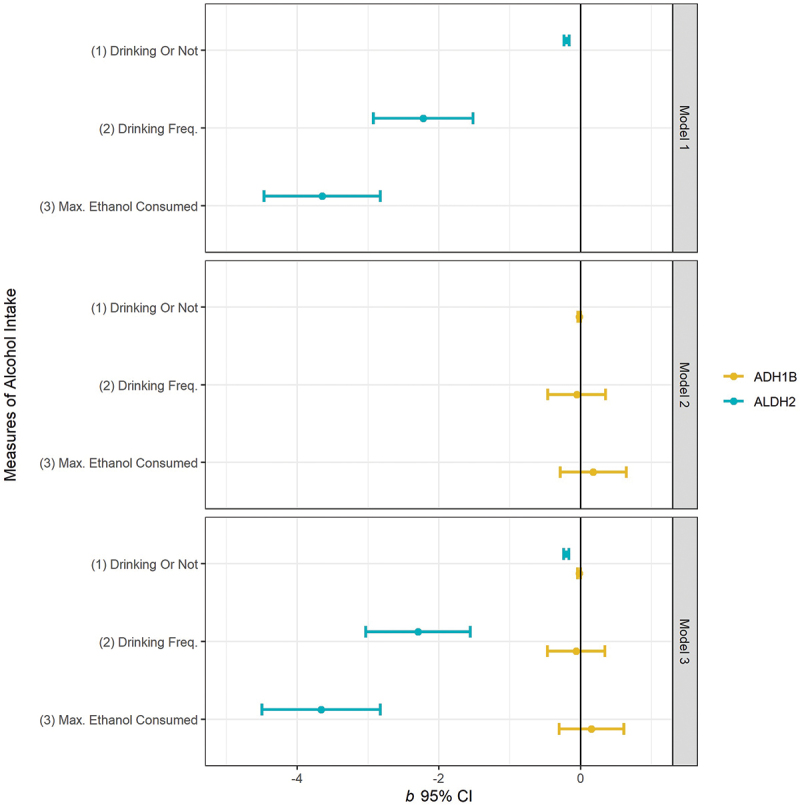
Associations between the *ALDH2* rs671/*ADH1B2* rs1229984 a alleles and different measures of alcohol intake.

Note: Plotted values are the beta coefficients [95% confidence interval (CI)] for carriage of an additional A allele derived from nine linear regressions. Model 1: control for *ALDH2* rs671 only. Model 2: control for *ADH1B* rs1229984 only. Model 3: control for both *ALDH2* rs671 and *ADH1B2* rs1229984. In all models, we control for male, age, educational attainment, annual income, father’s drinking habit, mother’s drinking habit, father’s genotype of *ALDH2* rs671, mother’s genotype of *ALDH2* rs671, province fixed effects and individual ancestry composition variables.

In population-based MR studies, the relevance condition for valid genetic IVs is usually satisfied *a priori*, given the robust association between the genetic variants of *ALDH2* rs671 and alcohol intake previously documented [29,48,49]. We verified that this condition also holds for our sample. Specifically, we first estimated the association between alcohol intake and the genetic variant of *ADH1B* 1229984, a genetic IV commonly used in MR studies conducted among European populations [25,27]. As shown in Figure 3, *ALDH2* rs671 statistically significantly predicts all measures of alcohol intake in our sample (Model 1). By comparison, the association between *ADH1B* 1229984 and alcohol intake (Model 2) is much weaker. Model 3 included both *ALDH2* rs671 and *ADH1B* 1229984 as explanatory variables. The strong association between *ALDH2* rs671 and alcohol intake persists, whereas *ADH1B* 1229984 is not significantly associated with any of the three alcohol intake measures at the 5% level. Nevertheless, because the use of multiple genetic IVs has been found to be promising in improving the precision of MR estimates [50], we included both *ALDH2* rs671 and *ADH1B* 1229984 as IVs in the MR estimation and formally tested for their joint significance in the first-stage regressions.

It is worth noting that there are very small differences in the magnitude of IV point estimates between using both genetic IVs (Model 3) and each IV separately (Models 1 and 2). This suggests that the two IVs are uncorrelated, as confirmed by a very low Pearson correlation coefficient of 0.0084 between them. The lack of correlation implies that using both IVs may add more power to the MR analysis and help improve estimation efficiency [51–53].

##### Independence: the genetic IVs must be uncorrelated with unmeasured confounders (conditional on other covariates)

**Figure A1.**
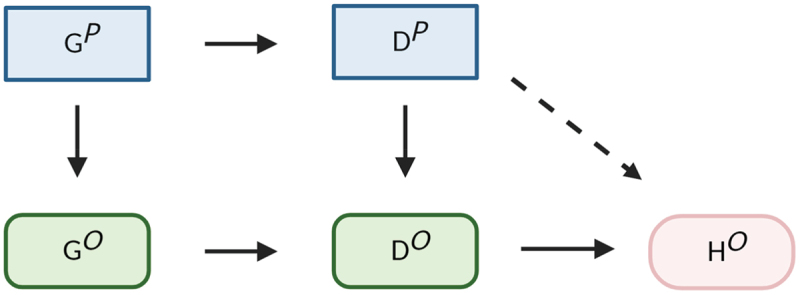
Directed acyclic graph (DAG) of potential dynastic effects. Note: DAG shows how dynastic effects may violate the independence assumption in an MR model. G^*P*^ is the parental genotype, D^*P*^ is parental drinking behaviour, G^*O*^ is offspring genotype, D^*O*^ is offspring’s drinking behaviour, and H^*O*^ is offspring’s health outcomes. The dashed line indicates the dynastic effect of the parents on offspring’s health outcomes, which introduces bias in MR estimates.

It has been recently recognized that the random inheritance of genetic variants from parents to offspring may not guarantee the independence between genetic variants and unmeasured confounders in a sample of unrelated individuals [32]. Two possible mechanisms may violate the independence assumption of genetic IVs: dynastic effects and population stratification [44,54,55]. Dynastic effects (depicted by the dashed line in Appendix Figure A1) refer to any indirect effects of parents’ genotypes on their offspring through parental phenotypes (also known as ‘genetic nurture effects’) [32,33]. For example, parents’ genotype will affect their drinking behavior, which may be correlated with their offspring’s genotype. If parental drinking affects offspring’s health outcomes but not through the latter’s alcohol consumption, this would violate the independence condition needed for valid genetic IVs. In this case, the detected effects may be due to the effects of parental alcohol intake on social or environmental factors that have impacted their offspring’s health outcomes. Such social or environmental transmission effects may contaminate the MR estimates.

Population stratification, on the other hand, refers to a systematic difference in allele frequencies or the presence of a specific health condition between subpopulations due to different ancestry [44,56–58]. A related methodological challenge is that, strictly speaking, genetic variants in MR designs are ‘as-if randomly assigned’ only when *conditional on* parental genes [44,59]. However, most existing MR studies on alcohol consumption failed to control for population stratification or dynastic effects that work through parental genes or individual ancestry, thus creating a potential threat: the genetic IV used may be correlated with unobserved ancestry and may lead to biased MR estimates [32,44,55].

We adopted two approaches to address potential dynastic effects and population stratification problems. The first was to include a total of 42 estimated compositions of individual ancestry based on each respondent’s genetic data as additional controls in the MR models (see the Appendix for further information). The second was to exploit the parent-offspring trios’ data on alcohol drinking-related traits and control for both parental drinking frequencies (i.e. D^*P*^ in Figure S1) and the inferred parental *ALDH2* rs671 genotypes (i.e. G^*P*^ in Figure S1) in the estimation. While our data contain no direct information on the respondents’ parental genetic markup, we were able to infer parental *ALDH2* rs671 genotypes based on (1) their phenotypes of alcohol flush reaction and (2) their offspring’s (i.e. the respondents’) precise *ALDH2* rs671 genotypes using Mendel’s laws of inheritance (see the Appendix for further information). The inclusion of parental phenotype and genotype variables of drinking closed the possible path of dynastic effects and thus strengthened the random assignment condition needed for a valid MR design [32,43,54].

##### Exclusion: the genetic IV must have no direct effect on health outcomes through horizontal pleiotropy

Horizontal pleiotropy occurs when a genetic IV influences multiple traits [33]. In our design, if the genetic variant of *ALDH2* rs671 or *ADH1B* rs1229984 directly affects the health outcomes of interest rather than indirectly through alcohol consumption, the exclusion condition for valid genetic IVs would be violated [26,44]. There are several reasons to believe that horizontal pleiotropy is not a threat to our MR design. First, Au Yeung et al. (2013) [47] and Peng et al. (2019) [29] have tested this assumption in a Chinese context and provided epidemiological evidence for the credibility of *ALDH2* rs671 as a genetic IV for alcohol intake. Second, we consulted with PhenoScanner (v2) [60] and found no evidence of direct links between *ALDH2* rs671 and *ADH1B* rs1229984 and allergy-related phenotypes. Third, we formally performed overidentification tests, which revealed no violation of the exclusion restriction (Appendix Table A1: Sargan statistics range between 0.030 and 2.388, with p-values ranging from 0.126 to 0.201). All these findings support the validity of *ALDH2* rs671 and *ADH1B* rs1229984 as genetic IVs for alcohol drinking in an MR setting.

#### Mendelian randomization estimates

Table 3 reports MR estimates of the effects of alcohol intake on allergic diseases for the full sample using both *ALDH2* rs671 and *ADH1B* rs1229984 as genetic IVs. Besides individual demographic/socio-economic characteristics, parental drinking behaviors, and province fixed effects that are commonly controlled for in existing MR studies, our MR models further control for inferred parental *ALDH2* rs671 genotypes and individual ancestral composition. Appendix Table A1 presents the corresponding first-stage estimation results, which indicate that *ALDH2* rs671 is robustly linked with lower levels of alcohol consumption. Moreover, the first-stage F-statistics for the joint significance of the IVs in all models ranged between 16 and 51, greatly exceeding the rule-of-thumb critical value of 10 for weak IVs [61], indicating that *ALDH2* rs671 and *ADH1B* rs1229984 can be jointly used as strong genetic IVs in our MR design. Moreover, Sargan statistics (ranging from 1.362 to 2.043) and the associated p-values (ranging from 0.153 to 0.243) from overidentification tests suggest no sign of the genetic IVs being correlated with unobserved confounders, further supporting the validity of these IVs.

**Table 3.** Mendelian Randomisation estimation results, full sample.

	(1)	(2)	(3)
	All (*N* = 3,041)		
Drinking or not	0.341***		
	(0.125)		
Drinking times during the past 30 days		0.026**	
		(0.011)	
Maximum number of standard drinks on one occasion			0.022**
			(0.010)
Age	−0.001	−0.003	−0.000
	(0.001)	(0.002)	(0.001)
Male	−0.034*	−0.049**	−0.071***
	(0.020)	(0.022)	(0.027)
Annual earnings	−0.001	−0.002*	−0.001*
	(0.001)	(0.001)	(0.001)
Years of schooling	0.002	0.008	0.003
	(0.005)	(0.005)	(0.005)
Mother drinks	−0.017	−0.023	0.001
	(0.021)	(0.023)	(0.019)
Father drinks	−0.059	−0.019	−0.018
	(0.039)	(0.035)	(0.035)
Mother: weighted number of A alleles	0.037*	0.042*	0.029
	(0.022)	(0.024)	(0.021)
Father: weighted number of A alleles	0.032	0.024	0.029
	(0.026)	(0.026)	(0.026)
Constant	−0.099	0.052	0.091
	(0.131)	(0.103)	(0.097)
Province fixed effects	Y	Y	Y
Ancestral controls	Y	Y	Y
Observations	3,041	3,041	3,041
First-stage F statistics	50.890	15.793	31.395
p-value	0.000	0.000	0.000
Sargan statistics	1.362	1.760	2.043
p-value	0.243	0.185	0.153

Robust standard errors in parentheses.

***significant at 1% level; **significant at 5% level; *significant at 10% level.

Compared with OLS estimates, an important takeaway from the MR estimates is that drinking is *not* causally associated with a lower risk of allergic diseases. Regardless of the specific measure used, a higher alcohol intake level is causally associated with higher risks of allergy in the full sample. Table 4 further presents MR estimates separately for male (columns 1–3) and female samples (columns 4–6), revealing sex-specific causal effects of alcohol consumption on allergy. In women, drinking [Table 4, column 4: *b* = 0.445, *p* = 0.032] and the number of drinking times during the past 30 days [Table 4, column 5: *b* = 0.031, *p* < 0.01] are causally associated with higher risks of allergic diseases. In men, however, little evidence of the causal impact of alcohol use on allergic diseases was found (Table 4, columns 1–3).

**Table 4.** Mendelian randomisation estimation results by sex.

	(1)	(2)	(3)	(4)	(5)	(6)
	Males (*N* = 1,826)			Females (*N* = 1,215)		
Drinking or not	0.237			0.445**		
	(0.154)			(0.207)		
Drinking times during the past 30 days		0.021			0.031***	
		(0.015)			(0.010)	
Maximum number of standard drinks on one occasion			0.014			0.025
			(0.009)			(0.024)
Age	−0.001	−0.003	−0.001	0.001	−0.000	0.001
	(0.001)	(0.003)	(0.001)	(0.002)	(0.002)	(0.002)
Male						
Annual earnings	−0.000	−0.001	−0.001	−0.002	−0.003	−0.002
	(0.001)	(0.001)	(0.001)	(0.001)	(0.002)	(0.002)
Years of schooling	0.002	0.010	0.002	0.004	0.006	0.010
	(0.005)	(0.007)	(0.006)	(0.009)	(0.008)	(0.009)
Mother drinks	0.027	0.014	0.033	−0.088**	−0.065*	−0.058
	(0.025)	(0.028)	(0.023)	(0.040)	(0.037)	(0.036)
Father drinks	−0.072	−0.054	−0.046	−0.024	0.064	0.060
	(0.045)	(0.041)	(0.040)	(0.073)	(0.062)	(0.062)
Mother: weighted number of A alleles	0.016	0.023	0.014	0.053	0.041	0.030
	(0.027)	(0.030)	(0.027)	(0.035)	(0.035)	(0.033)
Father: weighted number of A alleles	0.021	0.021	0.020	0.060	0.030	0.036
	(0.031)	(0.033)	(0.030)	(0.045)	(0.039)	(0.046)
Constant	−0.008	0.070	0.123	−0.322	−0.100	−0.156
	(0.158)	(0.123)	(0.111)	(0.220)	(0.175)	(0.178)
Province fixed effects	Y	Y	Y	Y	Y	Y
Ancestral controls	Y	Y	Y	Y	Y	Y
Observations	1,826	1,826	1,826	1,215	1,215	1,215
First-stage F statistics	31.176	13.875	23.036	19.397	12.414	15.968
p-value	0.000	0.000	0.000	0.000	0.000	0.000
Sargan statistics	0.030	0.035	0.459	2.372	2.443	2.388
p-value	0.864	0.852	0.498	0.124	0.118	0.122

Robust standard errors in parentheses.

***significant at 1% level; **significant at 5% level; *significant at 10% level.

## Discussion

Based on an MR design applied to a dataset of 3,041 individuals (1,826 men and 1,215 women) in China, this study demonstrated that, overall, a higher alcohol intake level is causally associated with a higher risk of allergic diseases in women (but not in men). Our MR results, obtained by using *ALDH2* rs671 and *ADH1B* rs1229984 as genetic IVs, showed that alcohol consumption can lead to allergic symptoms in Chinese females, and the observed negative or null association between drinking and allergy in conventional linear regression models is not causal. We verified the credibility of these genetic IVs by showing that the joint use of *ALDH2* rs671 and *ADH1B* rs1229984 satisfied the three core assumptions of valid IVs (i.e. relevance, independence, and exclusion restrictions) [26,43,44], warranting their usage in future MR applications.

It is worth noting that whether alcohol consumption will lead to allergic diseases is still under heated debate. Although observational analyses have reported that higher alcohol consumption is associated with allergic diseases, recent MR studies suggested that the link may not be causal [1,28,62]. On the contrary, our MR findings provided new evidence that the associations are indeed causal. One critical distinction between our MR analysis and previous MR studies is that we used both *ALDH2* rs671 and *ADH1B* rs1229984 as genetic IVs instead of only *ADH1B* rs1229984 as in existing MR studies [1,28]. A possible explanation of the differences between previous MR findings and ours is that the link between alcohol intake and *ADH1B* rs1229984 is much weaker than that of *ALDH2* rs671; hence, the use of *ADH1B* rs1229984 as the only IV may lead to a weak instrument bias [52]. In fact, the first-stage estimation results reported in Table A1 revealed a weaker association between *ADH1B* rs1229984 and alcohol intake. To further demonstrate this point, we re-ran all MR models but using *ADH1B* rs1229984 as the only IV. The additional results suggested a null effect of alcohol consumption on allergic diseases. Note that the first-stage F-statistics ranged between 2 and 6 (across different measures of alcohol intake), failing to exceed the rule-of-thumb cut-off of 10, flagging a weak instrument problem (detailed estimation results are available upon request). In contrast, the joint use of both *ALDH2* rs671 and *ADH1B* rs1229984 as genetic IVs in our study increased the statistical power and mitigated possible bias due to weak instruments, yielding more credible estimates of the effect of alcohol consumption [51–53].

Another main strength of our study lies in the relatively accurate control of parental genotypes of *ALDH2* rs671 and parental drinking habits. Theoretically, the random allocation of genotypes at meiosis, the underlying foundation of MR designs, is only satisfied when *conditional on* parental genotypes [63,64]. Our results are thus robust to possible bias initiated by assortative mating and dynastic effects.

Different from Cho et al. (2015) [23], who reported a very low level of alcohol consumption among South Korean females irrespective of the *ALDH2* rs671 genotypes (the percentage of current drinkers was 25.7%), the younger generation of Chinese women in our sample were observed to enjoy regular drinking (the percentage of current drinker being 86.5%) that is comparable to Chinese men (the percentage of current drinker being 88.7%). Yet, these divergent alcohol consumption patterns observed in two major East Asian countries may not be surprising because China’s drinking culture has switched from one predominantly with social and business drinking to one with more and more personal drinking, and China’s alcohol intake per capita is projected to surpass that of the United States by 2030 [65]. Such a growing trend raises further concerns about the adverse health outcomes and disease burden resulting from drinking in China. Our study adds to the literature on the alcohol-health nexus by showing that increased alcohol consumption is causally associated with higher risks of allergic diseases, at least in women.

Note that significant gender differences in the alcohol-allergy relationship identified were revealed in the MR analyses. These differences could be due to gender differences in the risks of allergic diseases. Some studies have found strong gender-specific risks of allergic diseases. For example, boys present allergic symptoms more often than girls in childhood, whereas women predominate allergic diseases in adulthood [66,67]. Given these findings, female respondents in our sample are subject to higher risks of allergic diseases (Table 1), as our sample consists of individuals over the age of 16. It is thus not surprising to see a more pronounced alcohol-allergy relationship among females in our sample.

Before closing, we note three caveats. First, the WeGene sample used in this study came mostly from urban China. More work would be required to confirm the external validity of our findings. Nevertheless, our analysis has strong internal validity, which is supported by the results of a series of robust and specification checks, and provides new evidence of the existence of a causal link between alcohol consumption and allergic diseases. Second, with 3,041 participants, our sample is relatively small, which may have limited our MR models’ power to detect the effect of alcohol intake in men due to the small sample size. Future studies that extend our study using large samples with genetic information are expected to be fruitful. Third, our study relied on self-reported data to construct the primary outcome variable (i.e. the incidence of allergic diseases), which may lead to discrepancies between the reported prevalence of allergies and that confirmed by clinical diagnosis. Future research may look for more objective and detailed measures of allergic diseases, such as asthma or allergic rhinitis, from clinical records. Ultimately, longitudinal datasets with both individual genotyping information and tracked health conditions would provide a deeper understanding of the long-term effects of alcohol consumption on allergic diseases.

## Data Availability

Data are available upon reasonable request. Deidentified participant data are available from the corresponding author (Qihui Chen) upon reasonable request and can be used only for research purposes. Reuse of the data should gain permission from the corresponding author of this article.

## Appendix METHODS

### Individual ancestry composition controls

Individual ancestry composition gives the percentage of DNA that comes from 42 different populations by comparing an individual genome to hundreds and thousands of people with known ancestry and is calculated using the ADMIXTURE program developed by the Department of Human Genetics, University of California Los Angeles.

The 42 ancestries are (from high to low): Northern Han, Southern Han, Mongolian, Japanese, Naxi/Yi, Dai, Gaoshan, Kinh, Korean, She, Tibetan, Tungus, Ashkenazi, Balkan, Bantusa, Bengali, Cambodian, Egyptian, English, Eskimo, Finnish/Russian, French, Hungarian, Iranian, Kyrgyz, Lahu, Mala, Mayan, Mbuti, Miao/Yao, Papuan, Pima, Sardinian, Saudi, Sindhi, Somali, Spanish, Thai, Uygur, Uzbek, Yakut, and Yoruba.

### Inferred parental ALDH2 genotypes

Alcohol flush (also known as Asian flush or Asian glow, symptoms include red flushing of the face or skin, headache, dizziness, nausea, etc.) has been found to be a phenotypic marker of the presence of A alleles in *ALDH2* rs671 gene (i.e. individuals with either the AA or AG genotype will exhibit the alcohol flush reaction after drinking, whereas individuals with the GG genotype are normal after drinking) [1,2]. Further, as a Mendelian trait, the alcohol flush phenotype resulting from *ALDH2* rs671 deficiency is independent of other genes or confounders [2,3]. Therefore, by combining information on whether parents exhibit alcohol flush and their offspring’s *ALDH2* rs671 genotype, we can calculate a weighted number of effect alleles that a parent carries (i.e. weighted by the probability of each possible genotype). For example, if both the father and mother of a respondent exhibit an alcohol flush after drinking, and the *ALDH2* rs671 genotype of this respondent is AA, then both of his/her parents must carry at least one effect allele (either genotype AA or AG, each occurring with 50% probability). Thus, the weighted number of A allele(s) that his/her parents have is calculated as $\overset{m}{\underset{n=1}{\sum }}\mathrm{Number}\;\mathrm{of}\;\mathrm{Aalleles}\;\mathrm{in}\;\mathrm{genotyp}e_{n}\cdot \mathrm{Probability}\;\mathrm{of}\;\mathrm{genotyp}e_{n}=2\cdot 0.5+1\cdot 0.5=1.5$. Similarly, if the father of a respondent does not exhibit alcohol flush while the mother does, and the respondent’s *ALDH2* genotype is AG, then his/her father must have a genotype of GG, while the mother is either AG (probability = 50%) or AA (probability = 50%), and the weighted numbers of A alleles are 0 and 1.5 for the respondent’s father and mother, respectively.

**References**

[1] Harada S, Agarwal DP, Goedde HW. Aldehyde Dehydrogenase-Deficiency as Cause of Facial Flushing Reaction to Alcohol in Japanese. Lancet. 1981; 2:982–982.

[2] Cho Y, Kwak S, Lewis SJ, Wade KH, Relton CL, Davey Smith G, Shin MJ. Exploring the utility of alcohol flushing as an instrumental variable for alcohol intake in Koreans. Scientific Reports. 2018;8(1):458.

[3] Brooks PJ, Enoch MA, Goldman D, Li TK, Yokoyama A. The alcohol flushing response: an unrecognized risk factor for esophageal cancer from alcohol consumption. PLoS Medicine. 2009;6(3):e50.

**Table A1.** First-stage estimation results.

	(1)	(2)	(3)	(4)	(5)	(6)	(7)	(8)	(9)
	All (*N* = 3,041)			Males (*N* = 1,826)			Females (*N* = 1,215)		
Variables	Drinking or not	Times of drinking, past 30 days	Maximum number of standard drinks, one occasion	Drinking or not	Times of drinking, past 30 days	Maximum number of standard drinks, one occasion	Drinking or not	Times of drinking, past 30 days	Maximum number of standard drinks, one occasion
ALDH2	−0.201***	−2.295***	−2.617***	−0.204***	−2.220***	−3.385***	−0.197***	−2.533***	−1.644***
	(0.020)	(0.409)	(0.332)	(0.027)	(0.566)	(0.499)	(0.032)	(0.591)	(0.364)
ADH1B	−0.021*	−0.062	0.109	−0.026*	−0.197	0.009	−0.022	0.259	0.267
	(0.011)	(0.225)	(0.181)	(0.014)	(0.301)	(0.265)	(0.019)	(0.344)	(0.208)
Male	0.017	0.755**	1.927***						
	(0.015)	(0.314)	(0.254)						
Age	0.002*	0.112***	0.019	0.003***	0.150***	0.037*	−0.002	0.026	−0.023
	(0.001)	(0.019)	(0.016)	(0.001)	(0.024)	(0.021)	(0.002)	(0.033)	(0.021)
Annual earnings	0.001**	0.054***	0.044***	0.001	0.042***	0.042***	0.002***	0.086***	0.045***
	(0.000)	(0.009)	(0.007)	(0.001)	(0.011)	(0.010)	(0.001)	(0.016)	(0.010)
Years of schooling	0.001	−0.212***	0.010	−0.002	−0.324***	0.020	0.006	−0.022	0.009
	(0.004)	(0.076)	(0.062)	(0.005)	(0.097)	(0.086)	(0.007)	(0.123)	(0.076)
Mother drinks	0.070***	1.012***	0.024	0.049**	0.973**	−0.283	0.101***	0.945*	0.545*
	(0.015)	(0.313)	(0.252)	(0.019)	(0.410)	(0.361)	(0.027)	(0.496)	(0.300)
Father drinks	0.123***	0.115	0.509	0.134***	0.628	0.892	0.124**	−0.965	−0.066
	(0.027)	(0.542)	(0.435)	(0.032)	(0.682)	(0.599)	(0.050)	(0.916)	(0.554)
Mother: weighted # of A alleles	0.025	0.006	0.502	0.019	−0.101	0.591	0.039	0.480	0.554
	(0.019)	(0.377)	(0.306)	(0.024)	(0.513)	(0.455)	(0.030)	(0.559)	(0.342)
Father: weighted # of A alleles	0.007	0.210	0.030	0.031	0.126	0.531	−0.029	0.167	−0.391
	(0.021)	(0.418)	(0.338)	(0.027)	(0.587)	(0.517)	(0.032)	(0.597)	(0.365)
Constant	0.748***	3.053**	1.176	0.783***	4.038**	2.549	0.712***	3.161	2.107
	(0.076)	(1.535)	(1.253)	(0.089)	(1.919)	(1.707)	(0.135)	(2.481)	(1.533)
Province fixed Eefects	Y	Y	Y	Y	Y	Y	Y	Y	Y
Ancestral controls	Y	Y	Y	Y	Y	Y	Y	Y	Y
Observations	3,041	3,041	3,041	1,826	1,826	1,826	1,215	1,215	1,215
R^2^	0.164	0.126	0.167	0.178	0.142	0.147	0.201	0.148	0.173

Notes: Robust standard errors in parentheses.

***significant at 1% level; **significant at 5% level; *significant at 10% level.
